# Strength and Microstructural Evolution of SRX-Stabilized Aeolian Sand–Gravel Flexible Base for Desert Road Construction

**DOI:** 10.3390/ma18173982

**Published:** 2025-08-25

**Authors:** Jie Liu, Qinli Liu, Chao Pu, Bo Wu, Xin Wang, Shiyu Zhu

**Affiliations:** 1 Xinjiang Transportation Planning, Survey and Design Institute Co., Ltd., Urumqi 830006, China; hfutliujie@163.com (J.L.);; 2 Xinjiang Key Laboratory for Safety and Health of Transportation Infrastructure in Alpine and High-Altitude Mountainous Areas, Urumqi 830006, China; 3 Key Laboratory of Transport Industry of Highway Engineering Technology in Arid Desert Areas, Urumqi 830006, China; 4 China State Construction Bridge Co., Ltd., Chongqing 402260, China; 5 College of Civil Engineering, Anhui Jianzhu University, Hefei 230009, China

**Keywords:** flexible base, SRX, aeolian sand, mechanical properties, microscopic analysis

## Abstract

This study investigates the strength and microstructural evolution of SRX-stabilized aeolian sand–gravel mixtures for flexible base applications in desert roads. CBR, UPS (uniaxial penetration strength), and compressive resilient modulus tests were conducted under varying SRX dosages (0.4–1.0%) and aeolian sand contents (30–50%). The results show that increasing the SRX dosage significantly improves all three indices, with the 0.5% SRX and 30% aeolian sand mixture yielding the CBR (385.89%) and UPS (0.938 MPa) and achieving a compressive resilient modulus that meets the requirements for graded aggregate base layers. XRD FTIR and SEM–EDS analyses reveal that the SRX enhances material structure primarily through physical mechanisms, forming dense films and bonding networks without inducing significant chemical reactions. Extended curing improves structural integrity, while excessive aeolian sand reduces compactness. SRX-stabilized aeolian sand gravel is a viable base and subbase material for desert highways.

## 1. Introduction

Semirigid base layers refer to those formed by stabilizing soil or granular materials with inorganic binders. With superior strength and rigidity, they provide excellent performance for the core load-bearing structures of highways. However, in practical applications, semirigid base layers face various issues, such as shrinkage and low toughness [1,2,3]. These problems can lead to surface cracks, rutting, or arch expansion [4,5]. In desert regions, these issues pose significant challenges for their use and maintenance of highways [6].

Furthermore, owing to the scarcity of aggregates in desert areas, improving the applicability of aeolian sand is a pressing technical problem for road construction in these regions [7]. Studies have shown that aeolian sand mainly consists of particles such as rock debris, feldspar, and quartz, and due to its non-plasticity and uniform particle size characteristics, it is a potential material for infrastructure [8,9,10]. Additionally, inorganic binders have been shown to strengthen aeolian sand to meet base layer specifications [11]. Scholars have also successfully used cement-stabilized aeolian sand for road construction in Jeddah [12]. Studies indicate that the 7-day unconfined compressive strength of an 11% cement-stabilized aeolian sand base meets high-grade road construction standards [13]. Moreover, fly ash-stabilized aeolian sand has a 28-day unconfined compressive strength of 1.56 MPa and a splitting strength of 0.16 MPa. Cement–fly ash-stabilized aeolian sand achieves a 7-day unconfined compressive strength of 2.8 MPa and a splitting strength of 0.61 MPa [14,15]. In summary, the stability of aeolian sand increases with increasing amounts of inorganic binder, but high dosages can lead to base damage [16,17].

Therefore, these issues can be addressed by introducing flexible base layers. Flexible base layers are typically made from graded aggregates or asphalt-stabilized materials [18]. The key advantage of these layers is their ability to effectively distribute the loads transferred from the surface structure, thereby extending the lifespan of the pavement [4]. Under standard loads, the lifespan of the flexible base layers is 1000 to 10,000 times longer than those of the semirigid base layers [19]. However, the strength of graded aggregate bases is closely related to the aggregate properties, gradation, and compaction. For example, the CBR strength of a dense gradation with 98% compaction is only 192.6% [20]. Even with high-quality aggregates, the CBR strength can only be slightly improved [21]. As a result, researchers have incorporated asphalt into the base stabilization process. Studies have shown that the strength of asphalt-stabilized base layers depends on the internal friction and cohesion; thus, compared with semirigid bases, they are superior in mechanical performance [22]. The compressive strength of asphalt-stabilized bases ranges from 7 to 8 MPa, which is similar to the 7-day strength of cement-stabilized bases, whereas the splitting strength ranges from 1.2 to 1.8 MPa, which is twice that of the cement-stabilized bases [19]. Additionally, compared with semirigid bases, asphalt-stabilized bases have better stress distribution performance and significantly superior crack resistance and rutting resistance [23]. However, due to the poor gradation and smooth particles of aeolian sand, its use alone cannot meet the load-bearing capacity requirements. A high asphalt content also increases costs. Therefore, exploring high-performance and cost-effective aeolian sand flexible bases has become a key focus of research.

At the end of the 20th century, Prinsloo successfully developed a new stabilizer, SRX (stable graded gravel road base polymer SoilFiX VR4). By adjusting its dosage, the base layer can exhibit either semirigid or flexible characteristics [24]. Research has shown that SRX can stabilize natural sands, graded gravel, and gravel soils to create flexible base layers suitable for road construction [25,26]. Compared with semirigid bases, SRX-stabilized bases have excellent performance in extending the road life, reducing costs, and protecting the environment [24,27,28]. For example, a 5% SRX-stabilized natural gravel base has a CBR of 227.85%, meeting the base layer requirements [29]. Similarly, a 0.5% SRX-stabilized graded gravel base achieves a CBR of 600%; this value is three times greater than that of a standard graded gravel base [30]. As a result, researchers have investigated key factors such as moisture content, aggregate gradation, SRX content, and compaction [31,32,33,34,35,36]. Zhang and Zhu showed the close relationship between the material performance and curing conditions [37,38,39]. Their results were consistent with Zhang’s findings on the impact of the moisture content on the strength of SRX-stabilized gravel bases [40]. Li and Zhao [41,42] reported that with increasing SRX dosage, the CBR strength, rebound modulus, and unconfined compressive strength initially increased.

However, traditional semirigid base layers in desert regions are prone to shrinkage and cracking, resulting in insufficient toughness and significantly compromising the service life of roads. At the same time, the strength of flexible base layers heavily relies on aggregate gradation and asphalt, making it difficult to substantially improve the CBR without increasing costs. Currently, research on SRX-stabilized base layers mainly focuses on gravel materials, while studies on their applicability in high-content aeolian sand gravel bases and the microstructural evolution of SRX remain lacking. To fill this gap, this study investigates the strength applicability of SRX-stabilized aeolian sand gravel flexible base layers under various SRX dosages and aeolian sand content. A series of tests, including the CBR test, uniaxial penetration strength test, and resilient modulus test, were conducted to verify that SRX-stabilized bases can still meet engineering requirements even with high aeolian sand content. Furthermore, the microstructural evolution of SRX at different curing ages was analyzed using FTIR, SEM, and EDS techniques, providing insights into its structural development and addressing the current research gap in this field.

## 2. Materials and Methods

### 2.1. SRX Polymer

The SRX used in the experiments was produced by Romix Industries Pty Ltd. (South Africa). It is a brownish liquid with a pungent odor. The experimental results meet the factory specifications [43,44]. The technical specifications are listed in Table 1.

**Table 1:** Physical and chemical properties of SRX.

Index	Test Result	Technical Requirement	Testing Methods
pH value	8	8–9	GB/T6920 [45]
Solid content/%	30.2	28–38	T0651 [46]
Viscosity/cps	80	50–100	ASTM D4486-91 [47]
Boiling point/°C	99	Approximately 100	GB/T616 [48]
Flammability	Non-flammable	Non-flammable	T0611 [49]
Specific gravity	1.02	>1.0	GB/T13531 [50]
Water solubility	Completely soluble	Completely soluble	GB/T259 [51]
Adhesion	Greater than 2/3	Coating area greater than 2/3	T0654 [49]

To investigate the main functional groups and microstructural morphology of the samples, FTIR and SEM tests were conducted. FTIR analysis was performed using the conventional KBr pellet method, with the primary components being the SRX solution and the dried SRX solid. The wavenumber range was set to 400–4000 cm^−1^ with a resolution of 4.000 cm^−1^. All measurements were carried out at room temperature to ensure coverage of the characteristic absorption regions of common molecular groups. For SEM analysis, the sample consisted of dried SRX solid blocks. Prior to testing, gold sputtering was applied using an ion sputter coater to enhance sample conductivity. The test was conducted at an accelerating voltage of 5.00 kV with a magnification of 3000×. Elemental mapping was performed for C, O, and Si, where C and O represent the main framework of the polymeric material, while Si and O are key components of the aeolian sand.

**Figure 1:**
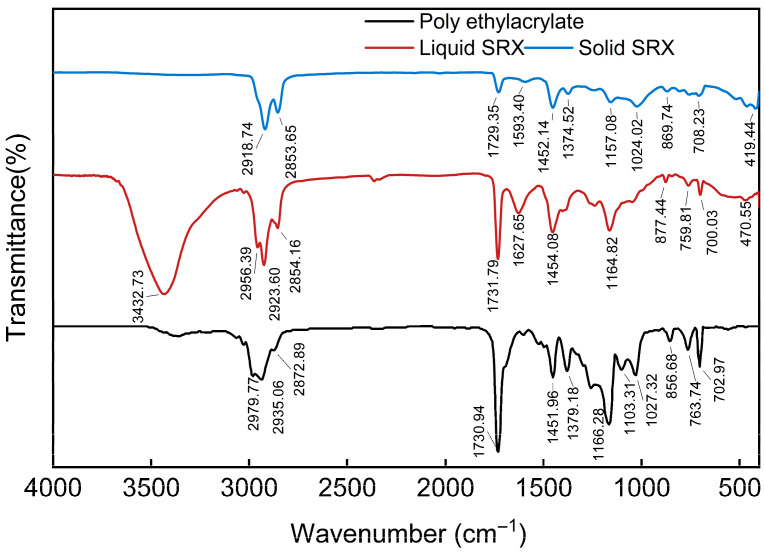
FTIR spectra of SRX and poly (ethyl methacrylate).

As shown in the FTIR spectrum (Figure 1), the liquid SRX solution exhibits a strong stretching vibration peak at 3432.73 cm^−1^, corresponding to the -OH group, indicating the presence of a large amount of water. Peaks observed at 2956.39 cm^−1^, 2923.60 cm^−1^, and 2854.16 cm^−1^ correspond to the C–H stretching vibrations, suggesting a substantial alkyl content in the liquid SRX, which is absent in the solid state. A strong absorption peak at 1731.79 cm^−1^ is attributed to the C=O stretching vibration, indicating the presence of ester groups. The C=C stretching vibration at 1627.65 cm^−1^ is characteristic of alkenes. Other major characteristic peaks remain largely unchanged between the liquid and solid states, suggesting that the SRX does not undergo chemical degradation with phase change. Furthermore, spectral matching via OMNIC software (version 8.2) reveals a high similarity (89.21%) to standard poly (ethyl methacrylate).In addition to typical peaks at 3000–2800 cm^−1^, 1730 cm^−1^, 1450 cm^−1^, 1380 cm^−1^, and 1300–1000 cm^−1^—associated with ester (C=O), methyl/methylene (C–H), and C–O–C linkages—SRX exhibits additional peaks below 1000 cm^−1^, indicating the potential presence of more complex chemical structures or functional groups. Overall, the main constituent of SRX is inferred to be poly (ethyl methacrylate), with the chemical formula –[CH_3_CCH_2_–COOCH_2_CH_3_]_n_–.

**Figure 2:**
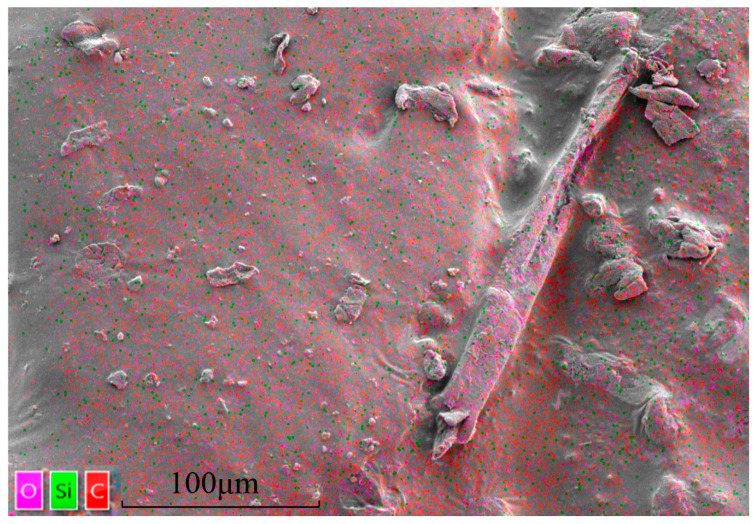
SRX micromorphology and element composition.

The SEM image of the SRX sample (Figure 2) shows that the fully dehydrated SRX forms a smooth and dense film-like morphology. This film structure exhibits a uniform surface and strong adhesion capability, effectively bonding fine aeolian sand and gravel particles, confirming the feasibility of using SRX as a stabilizing agent. Additionally, under high magnification, polymer fibers formed by tensile forces were observed on the film surface. These fibers exhibit stretched and curled morphologies, reflecting the good plastic deformation ability of SRX, which imparts flexibility to the stabilized material. EDS mapping results (Figure 2) further confirm that C and O elements are evenly and densely distributed on the surface, indicating that the surface is formed from the dehydrated SRX polymer. Notably, the red and purple element maps show clear contours around the fiber regions, while the green Si map shows no such signal, confirming that the O signal originates predominantly from the polymer rather than the aeolian sand. Nevertheless, the widespread distribution of Si indicates that fine sand and gravel particles are indeed adhered to the polymer surface.

### 2.2. Aeolian Sand

The aeolian sand was sourced from the Gurbantünggüt Desert, with its technical parameters listed in Table 2. The sand particles are light yellow and primarily composed of columnar, granular, and flaky particles. Laser diffraction particle size measurements indicate poor grading [52], with a high concentration of particles in the size ranges of 1.25 μm to 40 μm and 60 μm to 550 μm.

**Table 2:** Fine aggregate test results and technical requirements.

Test Item	Test Result	Technical Requirement	Testing Methods
Apparent density/g·cm^−3^	2.643	≥2.500	T0308 [53]
Organic matter content/%	1.35	≤10	T0336
Sulfate content/%	0.13	≤0.8	T0341
Natural moisture content/%	1.3	—	T0332
Particle analysis	Poorly graded	—	T0327

### 2.3. Crushed Gravel

The gravel used in the experiments was locally produced crushed gravel. The experimental results and technical specifications are listed in Table 3.

### 2.4. Overall Procedure

First, according to the stepwise filling method, gravel size fractions of 19.0–26.5 mm, 9.5–19.0 mm, and 4.75–9.5 mm were used in a mass ratio of 30:20:50. Based on the Romix Industries Pty Ltd. recommended SRX dosage of 0.5% and the results of preliminary experiments, the optimized mixture rations (as listed in Table 4) were selected for macro-scale mechanical performance and microstructural mechanism investigations.

**Figure 3:**
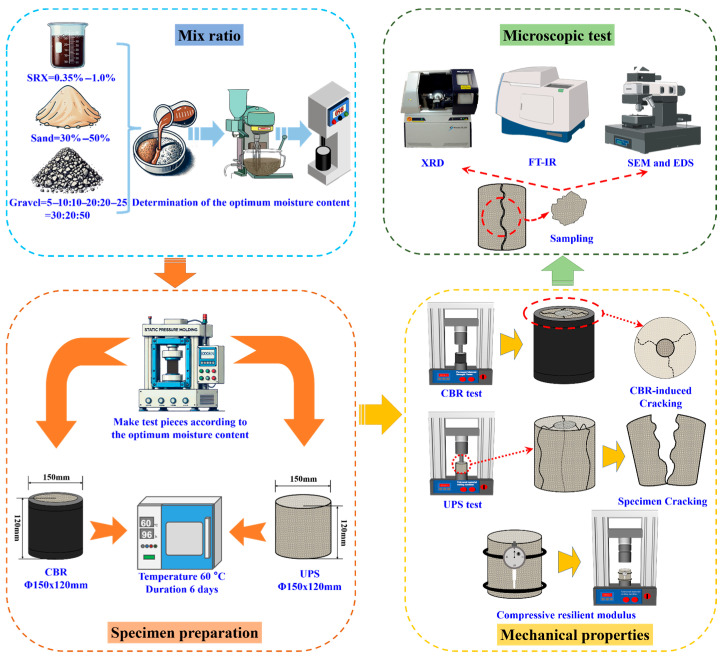
Technical workflow and main experimental procedure diagram.

At the macro level, the mechanical performance of SRX-stabilized aeolian sand–gravel flexible base layers was evaluated through CBR, UPS, and resilient modulus tests. At the micro level, XRD, FTIR, and SEM-EDS techniques were employed to analyze the development of material properties and elucidate the formation mechanisms. A comprehensive performance evaluation of the SRX-stabilized aeolian sand–gravel flexible base was ultimately conducted to support the engineering application of SRX and aeolian sand under special environmental conditions. The technical route and main experimental procedures are illustrated in Figure 3.

### 2.5. Test Methods

The mechanical performance of the SRX-stabilized aeolian sand–gravel flexible base was evaluated using the CBR and UPS. The CBR was conducted in accordance with the T0134-2019 [54]. The UPS followed the method outlined in Appendix F of the JTG D50-2017 [18]. According to the optimum moisture content and maximum dry density shown in Table 5, cylindrical specimens with dimensions of φ150 mm × h120 mm were prepared using a static compaction method.

**Figure 4:**
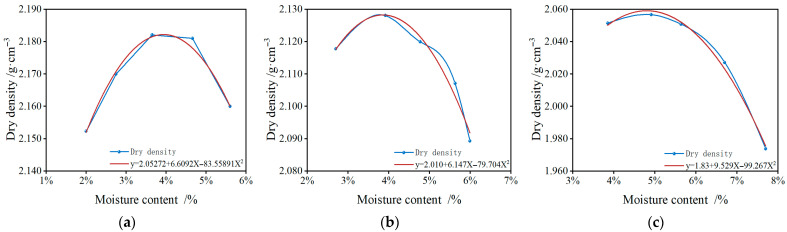
Optimum moisture content and maximum dry density. (**a**) 30% aeolian sand; (**b**) 40% aeolian sand; (**c**) 50% aeolian sand.

The optimum moisture content was determined based on the JTG 3441-2024 [55], using the T0804-1994 method [55] for the heavy compaction test. The gravel sample with a nominal maximum particle size of 26.5 mm was subjected to the “Method C” heavy compaction test, which included three compaction rounds with 98 blows each. After each round, the surface was scarified, and after all three rounds, the sample was weighed and dried at 105 °C to determine its final weight. Various water content levels were tested, and the relationship between water content and dry density was plotted (Figure 4). The optimal humidity was then calculated using linear fitting based on these experimental data.

After molding, the specimens were cured in an environmental chamber at 60 °C for six days and then tested using the GXYSG-127V pavement material strength tester (Zhejiang Chenxin Machine Equipments Co., Ltd., Shaoxing, China) for the CBR and the HUT305A microcomputer-controlled electro-hydraulic servo universal testing machine (Shenzhen Wance Technologies Ltd., Shenzhen, China) for the UPS.

The resilient modulus test was conducted in accordance with the T0808-1994 [55] test method. The specimen mix design, molding method, dimensions, and curing conditions were consistent with those used in the unconfined compressive strength (UCS) test. Based on the maximum load obtained from the UCS test, the preloading force was set to 0.5 F_m_. The applied loads were then incrementally divided into 0.1 F_m_, 0.2 F_m_, 0.3 F_m_, 0.4 F_m_, and 0.5 F_m_. Each load was applied and maintained for 60 s, during which the dial gauge displacement was recorded as L. The load was then reduced to 0.05 F_m_ and held for 30 s, during which the dial gauge reading was again recorded as L. After completing each loading–unloading cycle, the resilient modulus was calculated using Equation (1).

(1) $$E_{c}=\frac{Fh}{A\left(L_{\mathrm{bigin}}-L_{\mathrm{end}}\right)}$$

The compressive resilient modulus *E_c_* (MPa) was calculated using the following parameters: *F* is the applied load (N), *h* is the specimen height (mm), with a gauge length of 100 mm used in this test, and *A* is the bearing area of the specimen (mm^2^).

XRD was conducted on the S1A3 and S3A3 samples cured for 14, 28, and 60 days to analyze the phase composition of the material. FTIR was used to scan the S1A1, S1A2, S3A1, and S3A2 samples after curing for 6, 14, 28, and 60 days, in the wavenumber range of 400–4000 cm^−1^. SEM and EDS were used to perform a 2000× magnification scan on the S1A1, S1A2, S3A1, and S3A2 samples after curing for 6, 14, and 60 days. Through microanalysis, the polymer reactions and the microstructural areas were observed and analyzed.

## 3. Results

### 3.1. Mechanical Performance Analysis

#### 3.1.1. CBR Strength

**Figure 5:**
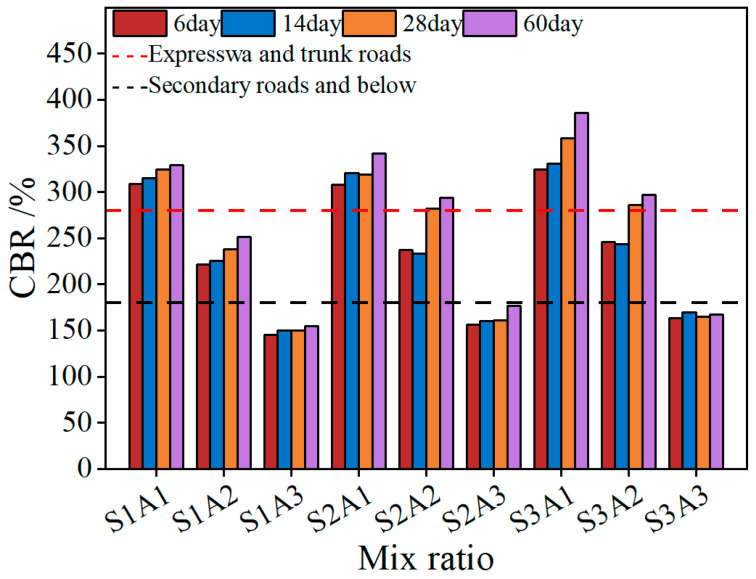
CBR with curing times.

The CBR test evaluates material strength by measuring the CBR under standard conditions [54], and it is a commonly used method for assessing the strength of flexible base layers composed of gravel or crushed stone. Figure 5 presents the CBR strength values of SRX-stabilized aeolian sand–gravel flexible base materials at different curing ages: 6 days, 14 days, 28 days, and 60 days.

During the strength development process, a general trend of increasing strength with longer curing periods was observed, reaching the maximum value at 60 days. This indicates that the SRX gradually dehydrates and consolidates over time, leading to a more stable structure. As the SRX dosages increased from 0.4% to 0.5%, the CBR strength showed a corresponding improvement. This is attributed to the enhanced bonding between aggregates induced by the SRX, which results in a denser structure and improved overall strength [56]. Consequently, the group with 0.5% SRX dosages cured for 60 days exhibited the highest strength, with S3A1 reaching a maximum CBR value of 385.89%. However, at a 50% aeolian sand content, the increase in CBR strength with curing age was not significant. Moreover, a comparison across different aeolian sand contents (30%, 40%, and 50%) revealed that higher aeolian sand content led to a decrease in CBR strength. This is because aeolian sand consists of finer particles, and excessive content reduces the interlocking effect among aggregates [57,58], thereby weakening the overall structural strength.

#### 3.1.2. UPS Strength

**Figure 6:**
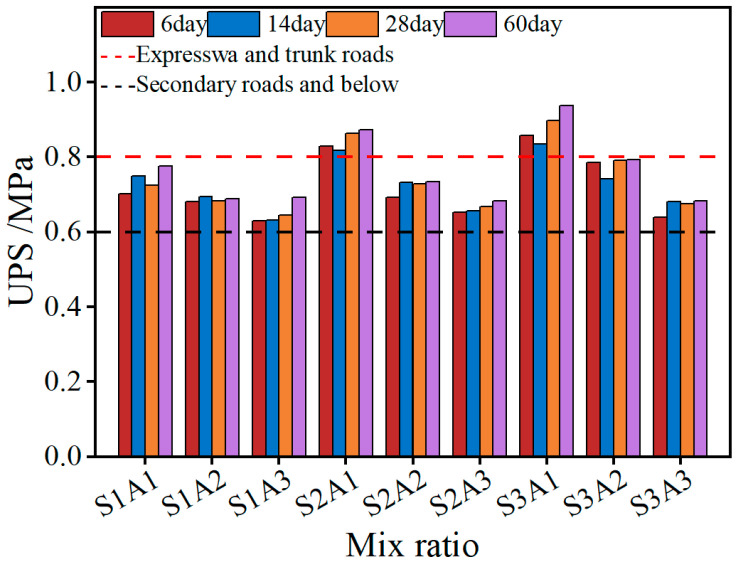
UPS with curing times.

Although the CBR value reflects the strength of granular materials, the stress characteristics and failure mechanisms of SRX-stabilized materials differ due to their combined interlocking and bonding effects. Therefore, following the approach proposed by Luo et al. [59], uniaxial penetration strength was adopted as an additional evaluation indicator for the SRX polymer-stabilized aeolian sand–gravel flexible base. As shown in Figure 6, the uniaxial penetration strength values of the stabilized base at different curing ages (6, 14, 28, and 60 days) exhibited slight increases or remained nearly unchanged across various mix designs.

This indicates that, in the absence of lateral confinement provided by molds, the strength of the material after dehydration and consolidation reaches a threshold. Hence, the bonding strength provided by the SRX polymer largely depends on the intrinsic properties of the polymer solution itself. This also explains why an increase in polymer dosage leads to an increase in material strength. Comparing different polymer dosages (0.4%, 0.45%, and 0.5%) reveals that the polymer enhances the bonding force between aggregates, resulting in a denser structure. Accordingly, the group with 0.5% polymer content exhibited the highest overall strength, with S3A1 reaching a maximum uniaxial penetration strength of 0.938 MPa after 60 days of curing. Furthermore, increasing the aeolian sand content was also found to reduce uniaxial penetration strength. Specifically, under the same polymer dosage, the group with 50% aeolian sand content had lower strength compared to the 30% and 40% groups. Unlike the CBR reduction, which is mainly due to reduced interlocking, this phenomenon is attributed to the smooth, rounded nature of aeolian sand particles. Excessive sand content reduces the bonding efficiency of the polymer, thereby weakening the overall strength.

**Figure 7:**
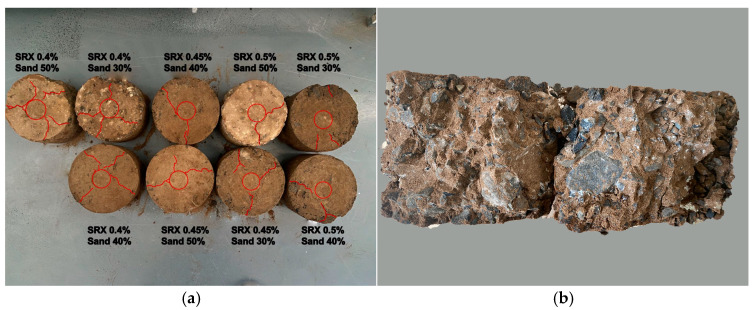
(**a**) Failure diagram after UPS with different mix ratios; (**b**) sectional view after UPS test.

Figure 7a illustrates the degree of specimen cracking after the uniaxial penetration test, helping to explain the strength variation trends under different mix proportions (where SRX denotes the polymer and Sand denotes aeolian sand).

With lower polymer content, specimens developed radial cracks on the top surface that extended through to the bottom, eventually breaking apart and losing strength. In contrast, with higher polymer content, radial surface cracks were significantly reduced, and the specimen typically split into two parts along a central fracture, as shown in Figure 7b.

### 3.2. Compressive Mechanical Response

#### 3.2.1. Ultimate Failure Stress

**Figure 8:**
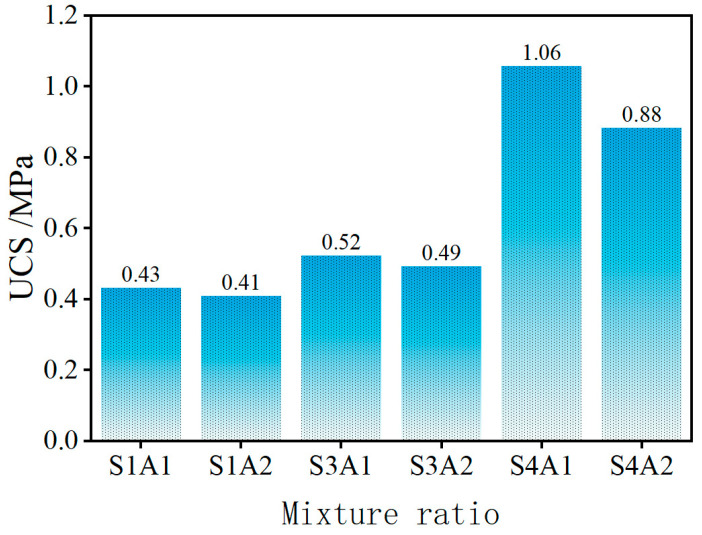
Ultimate failure stress.

As shown in Figure 8, the UCS of the SRX-stabilized aeolian sand–gravel flexible base exhibits a significant upward trend with increasing SRX and aeolian sand content.

Specifically, at 0.4% SRX, the UCS for aeolian sand contents of 30% and 40% are 0.43 MPa and 0.41 MPa, respectively. The difference is minimal and the overall strength is relatively low, indicating that the stabilized material behaves similarly to conventional granular flexible base materials. Moreover, under unconfined compressive conditions, the UCS enhancement provided by a low SRX dosage is limited, suggesting that UCS is not a suitable indicator for evaluating the performance of this type of material. When the SRX dosage is increased to 0.5% and 1.0%, the UCS rise to 0.52 MPa and 0.49 MPa, and 1.06 MPa and 0.88 MPa, respectively—representing increases of 20.93%, 19.51%, 146.51%, and 114.63% compared to the 0.4% SRX group. This demonstrates that higher SRX content significantly enhances the mechanical properties of the base material. However, aeolian sand content exhibits a negative correlation with compressive strength. This phenomenon can be attributed to the fact that, although higher SRX dosages improve bonding and material integrity, excessive aeolian sand may lead to a looser internal structure, thereby reducing strength. Among all mix designs, S4A1 achieved the highest compressive strength of 1.06 MPa, indicating that a 1.0% SRX dosage significantly enhances the material’s strength. This suggests that at higher dosages, the SRX not only improves the bonding between aeolian sand and gravel but also enhances the overall stability and compressive capacity of the base. Although S4A2, with a strength of 0.88 MPa, was lower than S4A1, it was still markedly higher than the other mix designs, indicating that even with increased aeolian sand content, a high SRX dosage can maintain strong material performance. In conclusion, the SRX dosage plays a decisive role in improving the strength of the aeolian sand–gravel flexible base. In particular, at a dosage of 1.0%, the strength performance is notably superior.

#### 3.2.2. Compressive Resilient Modulus

**Figure 9:**
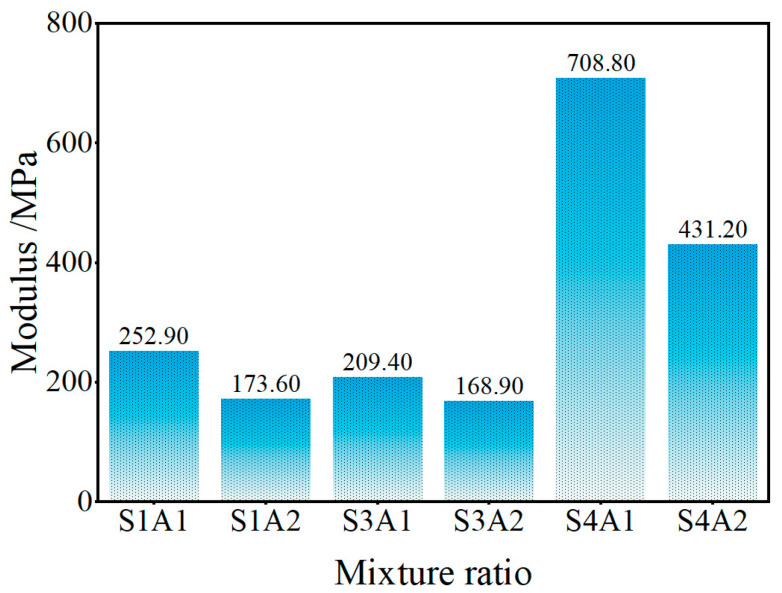
Compressive resilient modulus.

The compressive resilient modulus is a key parameter used to characterize the stiffness and behavior of flexible base layers. The calculated results are presented in Figure 9. Under SRX dosages of 0.4% and 0.5%, the resilient modulus remained relatively low with insignificant variation, indicating limited elastic recovery capacity of the material. To further verify the effect of SRX dosages, an additional group with 1.0% SRX (Group S4) was introduced. The results showed a marked improvement in resilient modulus at higher SRX dosages. In particular, the S4A1 mix achieved a resilient modulus of 708.80 MPa, significantly higher than the other groups. This demonstrates that increasing the SRX dosage substantially enhances the elastic behavior and structural stability of the material, confirming the positive role of SRX dosages in improving resilient performance. In contrast, the increase in aeolian sand content significantly reduced the resilient modulus. For the 0.4%, 0.5%, and 1.0% SRX groups, a 10% increase in aeolian sand content led to a decrease in compressive resilient modulus by 31.4%, 19.4%, and 39.1%, respectively. The substantial decline is primarily attributed to the fine and smooth texture of aeolian sand particles, which limits bonding strength and results in a looser internal structure. Under loading, such materials are more prone to deformation and exhibit poor elastic recovery, thereby reducing the resilient modulus. Additionally, higher aeolian sand content tends to increase the porosity of the mixture, which further contributes to greater compressive deformation and diminished recovery capacity under cyclic loading.

Nevertheless, the 0.4% SRX dosage group (i.e., S1A1 and S1A2) achieved resilient moduli of 252.90 MPa and 173.60 MPa, respectively, while the 0.5% SRX dosage group (i.e., S3A1 and S3A2) reached 209.40 MPa and 168.90 MPa. As shown in Table 6, these values are significantly higher compared to the resilient modulus of dry aeolian sand (19.09–41.48 MPa) and moist aeolian sand (27–76.71 MPa) [60].

They are also within or close to the typical ranges for graded crushed stone bases (200–400 MPa) and graded gravel (150–300 MPa) [18]. These results indicate that the incorporation of a SRX not only substantially enhances the resilient modulus of aeolian sand but also enables the stabilized material to meet the resilient modulus requirements of graded gravel bases, even with a high aeolian sand content. This demonstrates the favorable resilient performance of the SRX-stabilized aeolian sand–gravel flexible base.

### 3.3. Microstructural Mechanism Analysis

#### 3.3.1. XRD

**Figure 10:**
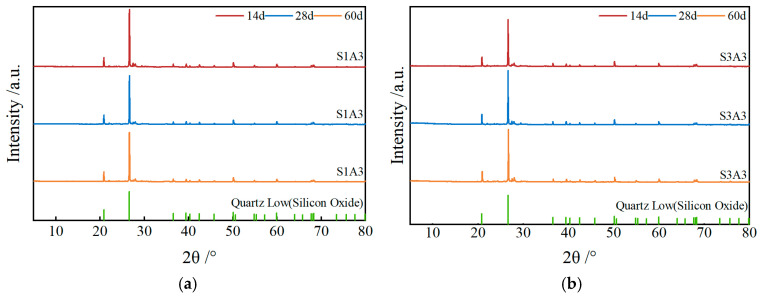
XRD at different curing ages: (**a**) 0.4% SRX; (**b**) 0.5% SRX.

As shown in Figure 10, XRD analysis of the SRX-stabilized aeolian sand–gravel flexible base material revealed that the primary crystalline phase is SiO_2_. Under SRX dosages of 0.4% and 0.5%, and at curing ages of 14, 28, and 60 days, the positions and intensities of the diffraction peaks remained unchanged, with no new peaks or significant changes in peak intensity observed.

This indicates that no notable inorganic chemical reactions occurred within the material during the curing process, and the crystalline phase structure remained stable. These results suggest that the primary function of the SRX is to enhance the structural performance of the aeolian sand–gravel mixture through physical stabilization rather than inducing chemical reactions.

#### 3.3.2. FTIR

**Figure 11:**
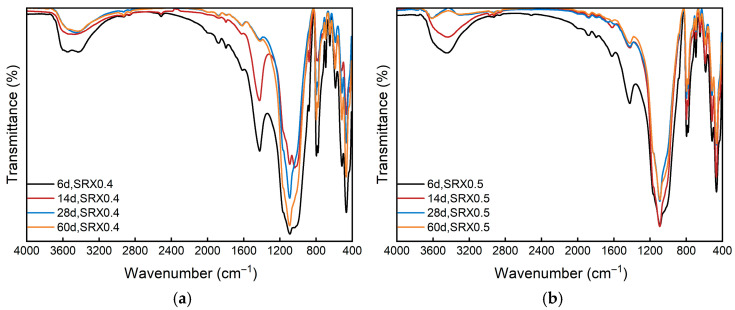
SRX FT-IR spectrum: (**a**) 0.4% SRX; (**b**) 0.5% SRX.

Figure 11 illustrates the variation in major characteristic peaks across the entire wavenumber range during the curing process for mixtures with 0.4% and 0.5% SRX dosages.

The region between 3500 cm^−1^ and 3400 cm^−1^ corresponds to the stretching vibrations of –OH groups, primarily associated with the presence of free water in the SRX solution. As shown in the spectra, the absorption intensity in this region gradually decreases as the curing time extends from 6 to 60 days. The reduction in free hydroxyl groups indicates a decline in free water within the sample, confirming that moisture loss and SRX solidification are key contributors to strength development. The weakening of the stretching vibration intensity around 1500 cm^−1^, corresponding to C=C bonds, suggests the completion of the SRX action reaction. Additionally, the characteristic peak near 1000 cm^−1^ is typically associated with the stretching vibrations of Si–O bonds, which primarily originate from the aeolian sand in the base material. As curing progresses, the surface of aeolian sand particles becomes increasingly coated by the SRX, reducing the vibrational freedom of Si–O bonds. Furthermore, the reduction in physical surface adsorption during early curing stages also contributes to the attenuation of Si–O absorption. In summary, the decline in FTIR peak intensities reflects the gradual stabilization of chemical interactions between the SRX and base material during the curing process, indicating the formation of a more consolidated and stable solidified structure.

#### 3.3.3. SEM and EDS

**Figure 12:**
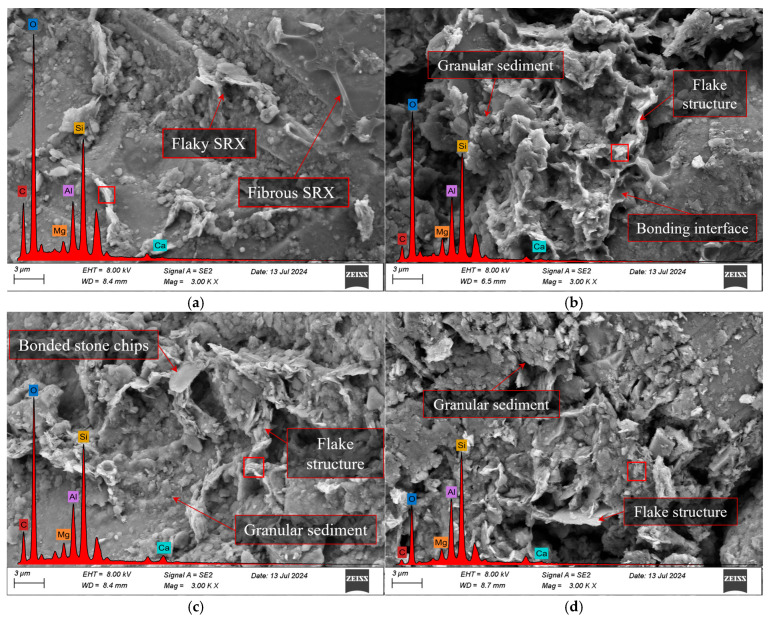
SEM images under 6-day curing conditions. (**a**) S1A1; (**b**) S1A2; (**c**) S3A1; (**d**) S3A2.

As shown in Figure 12 and Table 7, SEM and EDS analyses were performed on S1A1, S1A2, S3A1, and S3A2 specimens after 6 days of curing.

In these analyses, the carbon (C) element primarily originates from the SRX, while the silicon (Si) element mainly comes from the aeolian sand. Overall, increasing the SRX dosages from 0.4% to 0.5% significantly improved the microstructure of the material by enhancing the bonding strength between the SRX and mineral particles. In particular, the 0.5% SRX samples exhibited more compact lamellar structures and a better interfacial bonding network. In contrast, as the aeolian sand content increased from 30% to 40%, more granular deposits appeared within the sample, and the distribution of the polymer between particles became less uniform, resulting in reduced bonding performance—especially under the 0.4% SRX condition. In S1A1, the polymer was mainly distributed as flaky films and fibrous structures on the surface of aeolian sand particles and between them, but the overall coverage was limited and distinct voids remained between particles. The corresponding EDS results showed a carbon content of 26.50% and a relatively low silicon content of 21.52%, indicating that under lower aeolian sand content, the polymer occupied a larger proportion of the system and effectively participated in particle bonding, though it failed to form a dense overall structure. In S1A2, SEM images revealed that the aeolian sand particles were more tightly packed and localized flaky polymer coverage formed initial bonding interfaces. However, the continuity of the polymer phase was still insufficient. As the aeolian sand content increased, the carbon content dropped to 7.96%, while the silicon content rose to 31.93%, reflecting a significant increase in the proportion of the inorganic mineral framework in the observed area. For S3A1, the sample exhibited the densest and most uniform microstructure, with tightly integrated lamellar structures, resulting in a compact overall morphology. Although S3A2 still contained numerous flaky polymer structures, the excessive voids between particles weakened the overall bonding. As a result, the carbon content further decreased to 8.81%, while the silicon content increased to 42.51%.

**Figure 13:**
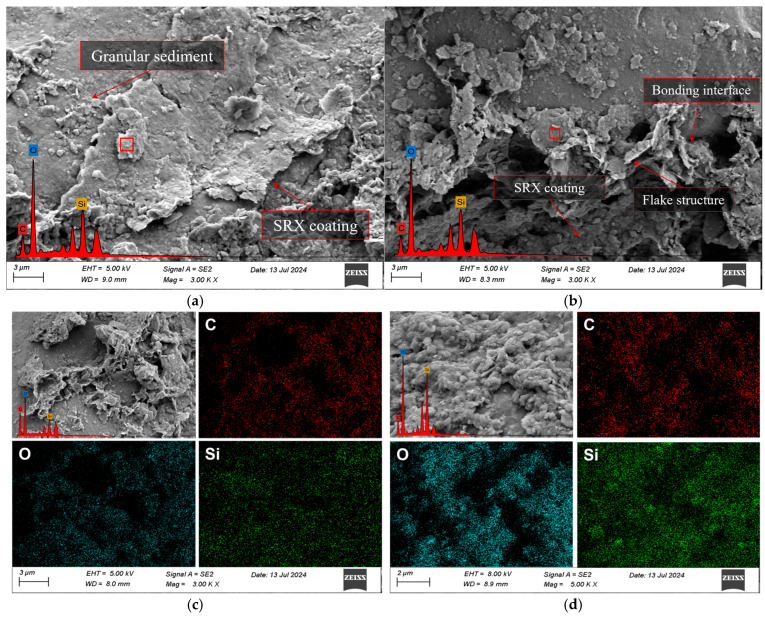
SEM images under 14-day curing conditions. (**a**) S1A1; (**b**) S1A2; (**c**) S3A1; (**d**) S3A2.

In the samples cured for 14 days, the structure exhibited noticeably greater compactness compared to those cured for 7 days. As shown in Figure 13, the 14-day cured mixtures developed more continuous bonding interfaces between particles, along with the presence of numerous thin film-like structures.

These films indicate that the water-based polymer had progressively coated the particles and formed an interwoven bonding network between them. The formation of these films results from further polymer solidification and its close adhesion to the particle surfaces, which contributes to enhanced material strength. In contrast, the 7-day samples showed poorly defined bonding interfaces and still contained many free voids between particles. According to the EDS mapping results in Table 8, the presence of C and O elements on the surface of the 14-day samples confirms that the material covering the particles is a polymer film, which significantly improves inter-particle bonding.

Moreover, as observed in samples such as S1A1 and S3A2, the particle surfaces in the 14-day cured specimens showed signs of being gradually wrapped and penetrated by the polymer, which helps improve the overall performance of the material. On the other hand, the microstructure of the 7-day specimens appeared rougher, with ineffective bonding between particles and insufficient surface coverage by the polymer. As a result, large voids and potential weak zones remained within the material. These voids can lead to stress concentrations under load, resulting in multiple cracking paths, as illustrated in Figure 7. The comparison of microstructure and elemental distribution between the two curing durations reveals that extended curing time is critical for fully activating the solidification function of the water-based polymer. This denser structure contributes to improved material strength, durability, and mechanical stability under loading.

**Figure 14:**
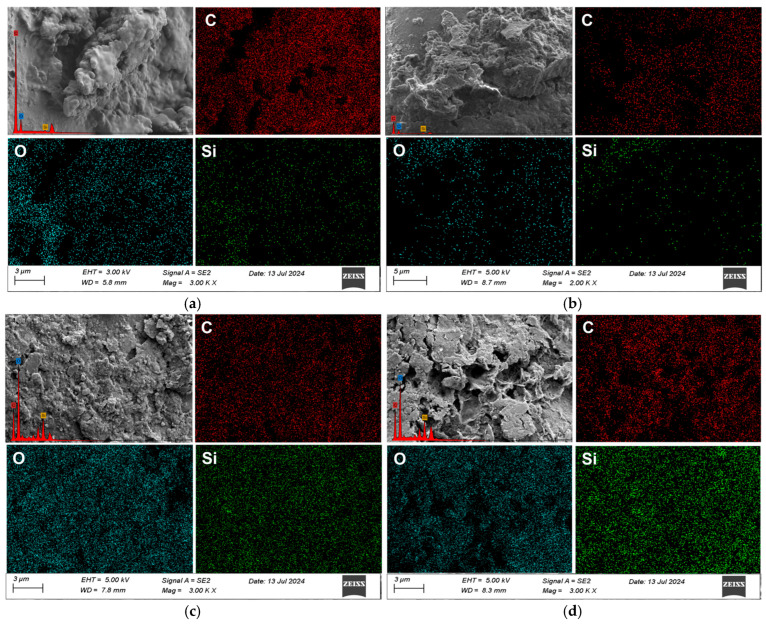
SEM images under 60-day curing conditions. (**a**) S1A1; (**b**) S1A2; (**c**) S3A1; (**d**) S3A2.

In the mixtures cured for 60 days, all mix designs exhibited significantly enhanced microstructural compactness and uniformity, as shown in Figure 14.

Voids between particles were nearly eliminated, and a stable film coating along with tightly bonded structures were observed. The particle surfaces were fully encapsulated by the polymer, with clearly defined bonding interfaces, and both chemical bonding and physical cross-linking between the polymer and the particles were evident. According to the elemental mapping results in Table 9, the distribution of C and O elements was uniform throughout the matrix.

In particular, the widespread presence of carbon indicates that the polymer had thoroughly infiltrated and firmly bonded to the entire matrix. The polymer network filled the inter-particle voids, forming a stable mesh-like structure. In the samples with 0.5% SRX, the extended curing duration allowed the polymer to fully penetrate and coat all particles, resulting in the near elimination of voids and the formation of highly stable bonding interfaces. This led to a densely cross-linked structure and superior mechanical performance. Although the samples with 40% aeolian sand content also showed relatively good compactness after prolonged curing, some bonding areas were less dense compared to the 30% aeolian sand samples due to the higher sand content. Therefore, prolonged curing time and increased SRX dosages significantly improved the infiltration and bonding performance of the polymer. Additionally, reducing the aeolian sand content further enhanced structural densification, ultimately improving the strength and durability of the stabilized material.

**Figure 15:**
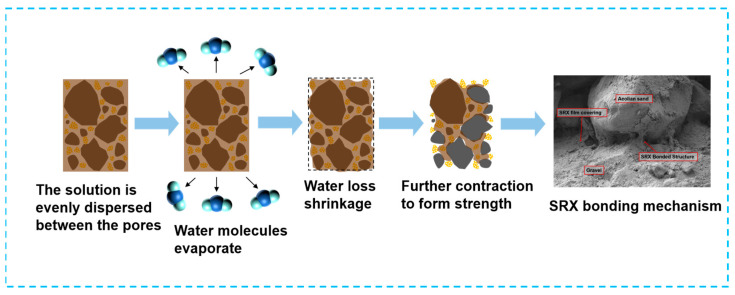
Strength growth mechanism of SRX-stabilized aeolian gravel base.

Figure 15 illustrates the strength formation mechanism of the SRX-stabilized aeolian sand gravel base.

Initially, SRX evenly fills the voids between the aeolian sand and gravel. As curing progresses, water molecules in the SRX evaporate, leading to consolidation, whereas the C=C double bonds are completely consumed, completing the polymerization reaction. Next, SRX undergoes consolidation and shrinkage to generate an initial bonding force between the aeolian sand and gravel; this results in a preliminary strength. Finally, as SRX fully reacts and water molecules are completely evaporated, the surface of the aeolian sand and gravel is tightly covered by a dense SRX film. Additionally, the bonding network structure formed between the aggregates significantly enhances the strength of the stabilized aeolian sand gravel base.

### 3.4. Feasibility Analysis

#### 3.4.1. Strength Suitability Study

The CBR and UPS standards of SRX-stabilized gravel are shown in Table 10 [61].

Table 11 presents the feasibility analysis of the aforementioned nine mix ratios, based on the requirements for different grades, loads, and purposes as specified in the standard [61].

#### 3.4.2. Economic Applicability Study

Based on Equation (2), the cost per square meter for a 1 cm thick layer was calculated, as shown in Table 12. The construction cost of the water-based polymer-stabilized base is 2.55 CNY/m^2^, which is lower than that of the asphalt-stabilized base (3.23 CNY/m^2^), but higher than that of the cement-stabilized base (0.34 CNY/m^2^). However, considering the limitations of cement-stabilized bases—such as poor crack resistance, limited durability, and high maintenance requirements—the water-based polymer exhibits a clear advantage in terms of cost-effectiveness. Not only does it achieve the structural performance of a full-depth flexible pavement, but it also offers substantial added value by shortening construction time and extending service life.

(2) Cost=XρCP/100

The cost per square meter was calculated using the following parameters: *X* is the thickness per square meter (cm); *ρ* is the density of the aggregate (t/m^3^), with an average value of 1.7 t/m^3^; *C* is the dosage of the stabilizer (%); and *P* is the unit price of the stabilizer (CNY/t).

## 4. Discussion

The mechanical performance results demonstrated that the increase in SRX dosage significantly enhanced the strength behavior of the flexible base, particularly in terms of CBR and UPS. This trend aligns with prior studies on SRX-stabilized graded crushed stone [29,37,38,59], where polymer film formation improves inter-particle bonding. However, in contrast to some cementitious stabilization mechanisms dominated by early-stage hydration [62,63,64,65], the strength improvement here was more gradual, suggesting that the dominant mechanism is physical solidification through water loss rather than chemical reaction. Furthermore, while most studies focus on well-graded gravel [35,40], this study validated the applicability of SRX in mixtures with high aeolian sand content. Although increased aeolian sand content reduced overall strength due to its smooth texture and reduced interlocking, the optimized 0.5% SRX–30% aeolian sand mixture still achieved strength levels comparable to conventional base materials, confirming its feasibility for desert road applications.

Regarding the microstructural mechanisms, the FTIR and SEM analyses in this study indicate that the primary enhancement mechanism is physical encapsulation and bonding by the SRX, rather than the formation of new crystalline phases. This is supported by XRD results showing no significant changes in diffraction peaks over curing time. In contrast, some previous studies [66,67] on cement- or lime-stabilized mixtures report noticeable mineralogical transformations such as the formation of C–S–H or ettringite, which significantly contribute to strength gain. The absence of such chemical reactions in this study highlights the distinct nature of SRX-stabilized systems, which rely predominantly on polymer-induced microstructural densification. This distinction underscores the need to adopt different evaluation approaches and design considerations when developing flexible bases using polymer-stabilized aeolian sand materials.

## 5. Conclusions

This study investigated the mechanical performance and microstructural evolution of SRX-stabilized aeolian sand–gravel mixtures for use in flexible base layers in desert road engineering. Based on a series of CBR, UPS, compressive resilient modulus tests, and XRD, FTIR, and SEM–EDS analyses, the following conclusions can be drawn:

1. The incorporation of a SRX significantly enhanced the strength and stiffness of the aeolian sand–gravel base. Even under high aeolian sand content, the stabilized mixtures achieved CBR values and resilient modulus levels that met or exceeded those of conventional graded gravel materials. The optimal performance was observed at a SRX dosage of 0.5%, where the CBR reached 385.89% and the UPS reached 0.938 MPa.
2. The SRX dosage significantly affected the resilient performance of the material. Under the combination of 1.0% SRX dosage and 30% aeolian sand, the compressive resilient modulus reached 708.80 MPa. Although increasing the aeolian sand content considerably reduced the modulus, the resilient modulus of the SRX-stabilized mixtures still met the standard requirements for graded gravel base layers.
3. XRD and FTIR analyses confirmed that the stabilization mechanism of the water-based polymer is primarily physical rather than chemical. The polymer forms a cross-linked network between particles without triggering significant inorganic reactions. The development of a dense, stable microstructure with curing time plays a key role in the performance improvement.
4. A higher aeolian sand content negatively affected mechanical performance and microstructural compactness. Due to the smooth, fine texture of aeolian sand particles, excessive content reduced aggregate interlock and polymer bonding efficiency, resulting in lower strength and stiffness. But increasing the polymer content from 0.4% to 0.5% or 1.0% substantially improved the material’s mechanical properties and microstructural integrity. SEM observations showed denser, more continuous film coverage and better particle encapsulation at higher polymer dosages. EDS analysis confirmed increased C element distribution, indicating effective polymer integration.
5. Based on the current research findings, future studies will focus on the durability and other pavement performance aspects of the SRX-stabilized aeolian sand gravel base layers under different environmental conditions.

## Figures and Tables

**Table 3:** Coarse aggregate test results and technical requirements.

Test Item		Test Result	Technical Requirement	Testing Methods
Apparent density/g·cm^−3^	19–26.5 mm	2.675	≥2.500	T0308
	9.5–19 mm	2.686		
	4.75–9.5 mm	2.686		
Crushing value/%		16.3	≤35	T0316
Los Angeles abrasion loss/%		17.6	<35	T0317
Soundness/%		5	<12	T0314
Flaky particle content/%		3.27	—	T0312

**Table 4:** Mixture ratios.

Mixture Ratios	SRX Dosage %	Aeolian Sand Content %
S1A1	0.4	30
S1A2	0.4	40
S1A3	0.4	50
S2A1	0.45	30
S2A2	0.45	40
S2A3	0.45	50
S3A1	0.5	30
S3A2	0.5	40
S3A3	0.5	50
S4A1 ^1^	1.0	30
S4A2 ^1^	1.0	40

^1^ S4A1 and S4A2 are supplementary mix designs for resilient modulus tests and are not included in the mechanical performance tests.

**Table 5:** Optimum moisture content and maximum dry density of different mix ratios.

Aeolian Sand Content	Optimum Moisture Content ^2^	Maximum Dry Density
30%	3.8%	2.183
40%	4.0%	2.128
50%	4.8%	2.059

^2^ SRX is a water-based polymer, and the optimum water content = SRX addition amount + diluted supplementary water.

**Table 6:** Resilient modulus range of granular materials.

Material Type and Layer Position	Compressive Resilient Modulus Range/MPa
Graded crushed stone base	200–400
Graded crushed stone subbase	180–250
Graded gravel base	150–300
Graded gravel subbase	150–220
Unscreened crushed stone layer	180–220
Natural sand–gravel layer	105–135
Dry aeolian sand	19.09–41.48
Moist aeolian sand	27–76.71

**Table 7:** EDS analysis after 6 days of curing.

Element	S1A1		S1A2		S3A1		S3A2	
	W_t_/%	A_t_/%	W_t_/%	A_t_/%	W_t_/%	A_t_/%	W_t_/%	A_t_/%
C	26.50	36.94	7.96	13.06	19.23	29.24	8.81	15.70
O	42.32	44.29	40.95	50.47	38.59	44.05	24.85	33.23
Mg	1.63	1.13	2.57	2.08	2.42	1.82	3.01	2.65
Al	7.18	4.45	15.95	11.65	9.20	6.23	19.01	15.08
Si	21.52	12.83	31.93	22.41	24.37	15.85	42.51	32.39
Ca	0.85	0.36	0.65	0.32	6.18	2.82	1.81	0.96

**Table 8:** EDS analysis after 14 days of curing.

Element	S1A1		S1A2		S3A1		S3A2	
	W_t_/%	A_t_/%	W_t_/%	A_t_/%	W_t_/%	A_t_/%	W_t_/%	A_t_/%
C	34.86	45.49	18.84	27.97	38.50	50.53	23.52	33.50
O	43.09	42.21	42.73	47.63	35.27	34.75	43.26	46.27
Si	22.05	12.30	38.43	24.40	26.22	14.72	33.22	20.24

**Table 9:** EDS analysis after 60 days of curing.

Element	S1A1		S1A2		S3A1		S3A2	
	W_t_/%	A_t_/%	W_t_/%	A_t_/%	W_t_/%	A_t_/%	W_t_/%	A_t_/%
C	86.94	90.43	74.03	80.98	32.52	42.93	36.83	47.97
O	11.17	8.73	19.43	15.97	44.45	44.06	40.00	39.11
Si	1.89	0.84	6.52	3.05	23.03	13.01	23.18	12.91

**Table 10:** CBR and UPS standards of SRX-stabilized gravel.

Structural Layer	Road Grade	Heavy and Very Heavy Traffic		Heavy Traffic		Medium and Light Traffic	
		CBR/%	UPS/MPa	CBR/%	UPS/MPa	CBR/%	UPS/MPa
Base layer	Expressways and primary roads	≥280	≥0.8	≥240	≥0.75	≥200	≥0.7
	Secondary and lower roads	≥240	≥0.7	≥220	≥0.65	≥180	≥0.6
Subbase layer	Expressways and primary roads	≥240	≥0.7	≥220	≥0.65	≥180	≥0.6
	Secondary and lower roads	≥220	≥0.6	≥180	≥0.55	≥140	≥0.5

**Table 11:** Feasibility analysis of SRX stabilization for aeolian sand and gravel.

Mix Ratio	CBR/%	UPS/MPa	Extremely Heavy, Heavy Traffic Highways and Primary Roads		Medium and Light Traffic Secondary and Lower-Level Roads	
			Base Layer	Subbase Layer	Base Layer	Subbase Layer
S1A1	308.86	0.703	—	√	√	√
S1A2	221.81	0.681	—	—	√	√
S1A3	150.12	0.630	—	—	—	√
S2A1	308.03	0.830	√	√	√	√
S2A2	237.11	0.692	—	—	√	√
S2A3	160.08	0.652	—	—	—	√
S3A1	324.28	0.857	√	√	√	√
S3A2	245.65	0.786	—	√	√	√
S3A3	169.32	0.639	—	—	—	√

**Table 12:** Table of economic evaluation.

Stabilization Method	Gradation Type	Dosage	Unit Price	Construction Cost
Cement Stabilization	C-B-1	5%	400 CNY/t	0.34 CNY/m^2^
Asphalt Stabilization	ATP-25	5%	3800 CNY/t	3.23 CNY/m^2^
SRX Stabilization	SRX-25	0.5%	30,000 CNY/t	2.55 CNY/m^2^

## Data Availability

The data that support the findings will be available in Figshare repository at https://doi.org/10.5281/zenodo.15805295 following an embargo from the date of publication to allow for commercialization of research findings.

## Abbreviations

The following abbreviations are used in this manuscript:

SRX	Stable graded gravel road base polymer SoilFiX VR4
CBR	California bearing ratio
UPS	Uniaxial penetration strength
UCS	Unconfined compressive strength
XRD	X-ray diffraction
FTIR	Fourier transform infrared spectroscopy
SEM	Scanning electron microscopy
EDS	Energy dispersive X-ray spectroscopy
E_C_	Compressive resilient modulus
